# Dog Y chromosomal DNA sequence: identification, sequencing and SNP discovery

**DOI:** 10.1186/1471-2156-7-45

**Published:** 2006-10-06

**Authors:** Christian Natanaelsson, Mattias CR Oskarsson, Helen Angleby, Joakim Lundeberg, Ewen Kirkness, Peter Savolainen

**Affiliations:** 1 School of Biotechnology, KTH, Royal Institute of Technology, AlbaNova University Center, 10691 Stockholm, Sweden; 2 The Institute for Genomic Research (TIGR), Rockville, MD 20850, USA

## Abstract

**Background:**

Population genetic studies of dogs have so far mainly been based on analysis of mitochondrial DNA, describing only the history of female dogs. To get a picture of the male history, as well as a second independent marker, there is a need for studies of biallelic Y-chromosome polymorphisms.

However, there are no biallelic polymorphisms reported, and only 3200 bp of non-repetitive dog Y-chromosome sequence deposited in GenBank, necessitating the identification of dog Y chromosome sequence and the search for polymorphisms therein. The genome has been only partially sequenced for one male dog, disallowing mapping of the sequence into specific chromosomes. However, by comparing the male genome sequence to the complete female dog genome sequence, candidate Y-chromosome sequence may be identified by exclusion.

**Results:**

The male dog genome sequence was analysed by Blast search against the human genome to identify sequences with a best match to the human Y chromosome and to the female dog genome to identify those absent in the female genome. Candidate sequences were then tested for male specificity by PCR of five male and five female dogs.

32 sequences from the male genome, with a total length of 24 kbp, were identified as male specific, based on a match to the human Y chromosome, absence in the female dog genome and male specific PCR results. 14437 bp were then sequenced for 10 male dogs originating from Europe, Southwest Asia, Siberia, East Asia, Africa and America. Nine haplotypes were found, which were defined by 14 substitutions. The genetic distance between the haplotypes indicates that they originate from at least five wolf haplotypes. There was no obvious trend in the geographic distribution of the haplotypes.

**Conclusion:**

We have identified 24159 bp of dog Y-chromosome sequence to be used for population genetic studies. We sequenced 14437 bp in a worldwide collection of dogs, identifying 14 SNPs for future SNP analyses, and giving a first description of the dog Y-chromosome phylogeny.

## Background

Population genetic studies of the earliest history of the domestic dog have so far mainly been based on the analysis of mitochondrial DNA (mtDNA), which represents a single genetic marker and, since it is maternally inherited, can only describe the history of females [1-4]. In order to have a second marker to corroborate the results based on mtDNA and to obtain a picture also for the history of male dogs, studies of the dog Y chromosome would be valuable. The Y-chromosomal genetic variation has been analysed in a few studies so far, but only for microsatellite variation [5,6]. Microsatellites have the disadvantage that mutations may be recurrent, rendering the phylogenetic relations between haplotypes not entirely clear. Single nucleotide polymorphisms (SNPs) on the other hand are an, in practice, digital form of information, since multiple mutations in the same nucleotide position is unlikely. It has been shown for humans that individuals may have Y-chromosomal haplotypes that are identical based on a large number of microsatellite markers even though the haplotypes as defined by biallelic markers are different, a contradiction which is due to recurrent mutations of the microsatellites [7]. This shows that Y-chromosomal biallelic markers are imperative for phylogenetic studies where they can serve as a backbone for the Y-chromosome phylogeny upon which the more detailed microsatellite variation can be imposed. Furthermore, for the dating of ancient population genetic events, analysis of DNA sequence variation offers a better tool than do microsatellites. There is thus a need for DNA sequence information for the dog Y chromosome.

However, so far only 3200 bp of nonrepetetive Y-chromosomal dog sequence has been reported in GenBank [8,9]. With the recent sequencing of the dog genome [10] the sequence for the autosomal and the X chromosomes were identified but, since the sequence was from a female dog, the Y chromosome sequence was not obtained. The genome of a male dog has earlier been surveyed by the sequencing with 1.5× coverage of a male dog [11], but because of the low coverage of this genome sequence an assembly into the different chromosomes is not possible and a definite identification of Y chromosomal sequences is therefore not possible.

In order to identify Y-chromosomal sequence we therefore compared the two genome datasets by Blast analysis, identifying sequences present in the male genome and absent in the female genome, and also compared the sequences with human Y chromosome sequence for further screening. The candidate sequences were then checked for male specificity by PCR analysis of male and female dogs. In order to identify SNPs to be used for population genetic studies we thereafter sequenced 14437 bp of the identified Y-chromosome sequence in 10 individuals representing dogs from all major populations around the world, giving a phylogenetic backbone for future population genetic studies of the Y-chromosome variation in dogs.

## Results and discussion

### Identification of Y chromosomal sequence

In order to identify Y-chromosome sequences in the male dog genome sequence we performed a combination of Blast and PCR analysis. For a sequence to be accepted as Y specific three criteria had to be fulfilled: (i) it should, in a Blast search against the human genome, have a best hit or be linked to a read with a best hit, to the human Y chromosome, (ii) it should in a Blast search against the reference female dog genome sequence have a negative result and (iii) it should in a PCR against samples from five male and five female dogs yield PCR product only for the male samples.

### Identification of candidate Y-chromosome sequences by Blast analysis

The Blast analysis was performed in two steps; to reject the thousands of male sequences that lack identity with the female genome sequence owing to gaps in the female genome assembly or polymorphisms between the two genomes, a screen of the male dog genome sequence against the human Y chromosome sequence was done before comparing with the female dog genome sequence. Assembled survey-sequence data, based on approximately two million contigs and singleton reads, from the genome of a male poodle [11] was compared to the reference human genome sequence using blastn. For 32 contigs, the highest-scoring alignments were with a single region on the human Y-chromosome (Additional file 1; rows 1–32). Component reads from these contigs were linked physically to mate reads from the other end of the insert in the same clones and these were, in turn, often part of distinct contigs consisting of several other reads. These sequences were also considered as possible Y-chromosome sequence (Additional file 1; rows 33–93) giving totally 93 potential Y-chromosome sequences which were further compared to the reference female boxer genome sequence [11]. Sequences sharing >95% nucleotide identity for more than 90% of their lengths with regions of the boxer genome sequence were discounted, resulting in 43 remaining sequences considered as potential Y-chromosome sequences (Additional file 1; Boxer Hit:"-").

### Confirmation of male specificity by PCR analysis

The 43 sequences considered potential Y-chromosomal sequence were tested for male specificity by PCR in two steps, first against a panel of two male and two female dogs (DNA sample panel 1), and the sequences passing this screen were further tested against a panel of three male and three female dogs (DNA sample panel 2). To investigate the correctness of excluding sequences with match to the female genome sequence, even though they had a best hit to the Y chromosome in comparison to the human genome, 29 of the sequences excluded based on this criterion were also tested. Upon agarose gel electrophoresis, sequences giving bands or smears of the same size for both males and females were excluded from further analysis, as were sequences giving no bands at all. Sequences giving bands only for males were kept as candidates.

35 sequences, of the 43 considered potentially Y-chromosome specific, passed the first PCR screen (Additional file 1). Of the eight sequences which did not pass, seven gave bands or smears for both males and females and one gave no bands at all. Of the 29 sequences excluded based on match to the female genome all failed, 28 giving both male and female bands and one giving no bands at all. The 35 sequences that passed the first screen were selected for the second PCR screen against three more male and female dogs to confirm the male specificity. 32 of the 35 candidates were approved (giving bands of correct size for all five males, and no band at all for all five females) and thus considered male specific. Of the three failed sequences two had a band for only one of the male dogs and the third one had a weak band for one female dog. The GenBank Accession number for these sequences, totally 24159 bp in length, can be found in Additional file 1.

### Sequence variation

In order to study the genetic variation for the Y-chromosome among dogs, and to identify nucleotide diversity to be used in population genetic studies, we performed DNA sequencing of 10 male dogs for 24 of the sequences identified as male specific, for a total of 14437 bp. Twelve of these sequences formed six pairs obtained from the same shotgun clone, with intervening unknown sequence. This sequence was determined through primer walking. The sequence data generated for the ten samples, including the here defined intervening sequence, have been deposited in GenBank, under accession numbers DQ973626–DQ973805. In order to avoid geographical bias for the identified polymorphisms and to cover a large amount of the genetic variation among dogs globally, the 10 dogs were chosen to represent several different populations and types of domestic dogs from all continents except Australia (DNA sample panel 3).

**Figure 1:**
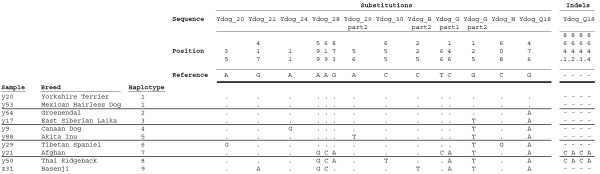
**Sequence alignment showing base substitutions and indels for the 10 analysed samples**. Only variable sites are shown, with sequence positions given above. Identity with the reference sequence (Yorkshire Terrier) is denoted by a stop, substitution by a different base letter, and deletion by a dash. Positions are denoted relative to the sequences submitted to GenBank (DQ973626 – DQ973805).

Among the 10 dogs, 9 haplotypes were found which were defined by a total of 14 polymorphic positions, 13 substitutions and 1 indel (Figure 1). As expected for haploid sequence, none of the individuals were heterozygous for any nucleotide position. The average number of differences between two individuals was 4.2, giving a π value of 2.91 × 10^-4^. A π value for the wolf Y chromosome of 0.4 × 10^-4 ^has earlier been reported [12], seven times lower than the here reported value for dogs. The reason for this discrepancy is not clear. One explanation might be that the here analysed dog sequences probably mostly derive from non-genic regions while the wolf sequences were mostly intronic sequence, possibly giving different substitution rates for the two data sets. Another explanation might be a geographical bias for the wolf sample (30/36 animals were from Russia (unspecified region)/the Baltics/Scandinavia, which because of the great mobility of wolves may be quite closely related populations). It can be noted that for the human Y chromosome π = 1.51 × 10^-4 ^(2.3 Mb of the NRY sequenced) [13], which is in best agreement with the value for the dogs in this study.

### Phylogenetic relationships

**Figure 2:**
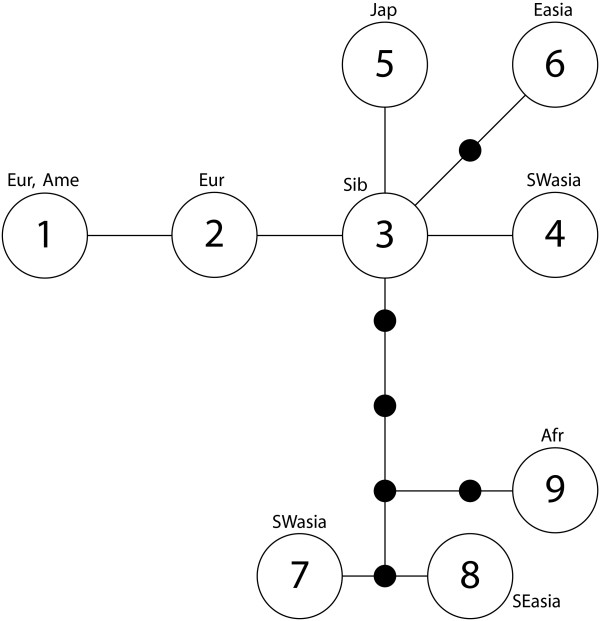
**Maximum parsimony phylogenetic tree of the nine haplotypes found in the sequence analysis**. Sequence types (circles) and empty nodes (solid dots) are separated by one mutational step (substitutions; indels are not shown).

The fact that analysis of ten individuals resulted in nine haplotypes indicates that there are numerous more Y-chromosome haplotypes to be found in the global dog population. A phylogenetic tree displays the relationship between the nine haplotypes (Figure 2). There is no clear geographic pattern for the distribution of haplotypes; possibly the sample is too small to show any such trends. The nucleotide substitution rate for the human Y chromosome is approximately 1.65 × 10^-9 ^per bp and year [14], and it has been shown that the average genome mutation rate is approximately constant among mammals [15]. Assuming a mutation rate of 1.65 × 10^-9 ^substitutions per year and bp also for the dog Y chromosome, in the lack of a species specific rate, we get a rate of one substitution per 42000 years for the analysed region. Assuming that the domestic dog originated around 15,000 years ago [2,16] this suggests that the nine dog Y-chromosomal haplotypes, several of which are more than one mutation step from its nearest neighbours, would originate from at least five wolf haplotypes. This agrees with an origin from a minimum of six female wolves according to mtDNA studies [2] and of totally a minimum of 21 individuals according to a study of dog MHC-variation [17].

## Conclusion

We have in this study, using Blast analysis and PCR screening among male and female dogs, identified 24159 bp of dog Y-chromosome sequence to be used for population genetic studies. We sequenced 14437 bp of this sequence in a worldwide collection of 10 dogs, giving a first glimpse into the dog Y-chromosome phylogeny. Nine haplotypes were found among the ten dogs, indicating that there are numerous more Y-chromosome haplotypes to be found in the global dog population. The 14 identified SNPs can be used for SNP analyses, and the SNP based phylogeny will serve as a backbone for studies of Y chromosome microsatellite variation.

## Methods

### Blast analysis of genome shotgun sequences

Assembled survey-sequence data from the genome of a male poodle [11] was searched against the reference human genome sequence [18] to identify sequences with a best match to the Y chromosome, using NCBI-blastn with the following parameters; -e 1e-3 -q -11 -r 10 -G 20 -E 10 -X 150 -F T -b 1 -v 1 -m 8. Sequences with several matches were excluded as repetitive sequences. The male poodle sequences were also searched against the reference female boxer genome sequence [19] using NCBI-blastn with the following parameters; -W 40 -b 1 -v 1 -m 8. A match was defined as an alignment with >95% nucleotide identity for >90% of the length of the poodle sequence, and sequences without a match were considered to be potential Y-chromosome sequences (Additional file 1).

### PCR analysis

#### DNA samples

Samples used for the first screening analysis for male specificity (Panel 1, blood samples): Males; German Sheppard (region of origin: Europe) and Doberman Pincher (Europe), Females; Toy Poodle (Europe) and Springer Spaniel (Europe). Samples used for the second screening analysis for male specificity (Panel 2, buccal epithelial cell samples): Males; Leonberger (Europe), Tibetan Spaniel (East Asia) and Samoyed (Siberia), Females; Leonberger (Europe), Chow-Chow (East Asia) and Thai Ridgeback (Southeast Asia). Samples used for sequence analysis (Panel 3, all males and buccal epithelial cell samples): Yorkshire Terrier (Europe), Belgian Groenendael (Europe), Caanan dog (Southwest Asia), Afghan Hound (Southwest Asia), East Siberian Laika (Siberia), Tibetan Spaniel (East Asia), Thai Ridgeback (Southeast Asia), Akita Inu (Japan), Basenji (Africa) and Mexican Hairless Dog/Xoloitzquintle (America).

#### DNA extraction

Blood samples were treated with heparin or EDTA. The DNA extractions from blood were performed using the protocol #PT 3628-1, version #PR 22673, for the "NucleoSpin Blood Kit" (Biosciences Clontech). Prior to PCR, extracted DNA from the blood samples was diluted by a factor of eight. Buccal epithelial cell samples were collected using Whatman FTA-indicating cards according to the manufacturer's specifications [20].

#### DNA amplification for test of male specificity

PCR amplification of target sequences was performed using a forward and reverse primer (Additional file 2). The PCR mixture consisted of 2 mM MgCl_2 _(Invitrogen), 20 mM TRIS-HCl pH 8.4 (Invitrogen), 50 mM KCl (Invitrogen), 200 mM of each dNTP (GE Healthcare), 1 unit PlatinumTaq DNA Polymerase (Invitrogen) and 200 mM of each primer in a total volume of 50 μl. The DNA template was 1 μl DNA extract from blood samples or a ∅ 2 mm piece from an FTA indication card. The reaction was run in a ThermoHybaid MBS 0.2 S (Thermo Electron Corporation) and the PCR program consisted of a predenaturation step (94°C, 2 min), followed by 40 cycles of denaturation (94°C, 30 sec), primer annealing (55°C, 30 sec) and extension (72°C, 3 min) followed by a final extension step (72°C, 10 min). The presence of amplification products was checked by analyzing five μl PCR product with agarose gel electrophoresis and staining with ethidium bromide.

#### DNA amplification for sequence analysis

PCR amplification of target sequences was performed in a nested configuration to improve specificity of the amplification (Additional file 2). Both outer and inner PCRs had PCR mixtures and PCR programs identical to the amplification for test of male specificity, with the exceptions that the template for the inner PCR was 0.5 μl of PCR product from the outer PCR and the number of cycles for the outer and inner PCR was, respectively, 15 and 35.

#### DNA sequence analysis

All nucleotide positions were sequenced by at least one forward and one reverse sequence read, using the primers given in Additional file 2. For the cycle sequencing reaction, 1 μl of the inner amplification product was mixed with 13 μl 1× cycle sequencing buffer (26 mM Tris, pH 9.0, 6.5 mM MgCl_2_), 1 μl big dye terminator (ABI Prism Big Dye Terminator Cycle Sequencing Ready Reaction Kit v2.1, Applied Biosystems) and 0.25 μM primer, for a total reaction volume of 20 μl. The reaction was run in a ThermoHybaid MBS 02 S. The cycle sequencing program consisted of 30 cycles of denaturation (96°C, 10 s), primer annealing (55°C, 15 s) and extension (60°C, 4 min). The cycle sequencing products were ethanol precipitated and analysed on an ABI 3700 according to the manufacturers' directions (Applied Biosystems). The DNA sequences were edited using Sequencing Analysis 2.1.1 (Applied Biosystems) and assembled into contigs and further edited in Sequencher 4.1 (Gene Codes Corporation). Alignment and comparison of sequences were performed in Sequencher 4.1.

## Authors' contributions

CN designed and carried out PCR screening, performed phylogenetic analysis and drafted the manuscript. MO designed sequence analysis and performed DNA sequencing. HA designed and carried out PCR screening and performed DNA sequencing. JL participated in co-ordinating and planning the study and helped drafting the manuscript. EK designed and performed Blast analysis and helped to draft the manuscript. PS conceived the study, and participated in its design and coordination and drafted the manuscript. All authors read and approved the final manuscript.

## Supplementary Material

Additional file 1 **Identification of Y-chromosome sequences; results from blast analysis and PCR screen**.
^a ^Name of shotgun sequence.
^b ^GenBank accession number of shotgun sequence used as template for primer design.
^c ^Best hit versus Human genome. Shown here is the highest-scoring alignment with a single region on the human genome.
^d ^Female dog genome hit. A match versus the female dog genome (sequences sharing >95% nucleotide identity for more than 90% of their lengths with regions of the female dog genome sequence) is indicated with a "+" and a non-match is indicated with a "-".
^e,f ^Result from the first (two males and two females) and second (three males and three females) PCR screen. "Candidate" indicates a sequence that gave a PCR fragment for male dogs only, "Not male specific" a sequence that gave a PCR fragment for both male and female dogs, a dash indicates a sequence that was not tested, "Approved" indicates a sequence that gave a PCR fragment for all five male dogs tested and no band for the five female dogs, and "Failed" indicates a sequence that did not give three male bands and/or gave female bands in the second test. Click here for file

Additional file 2 **Primers used for PCR and sequence analysis and positions analysed in DNA sequencing**.
^a ^Name of analysed sequence. GenBank accession number given in Additional file 1. The shotgun sequencing approach yields two sequences for each clone, one from each end of the insert. Such a pair can be analyzed as a single unit in the PCR screen (if the intervening sequence is short enough to allow a PCR). Twenty sequences, among the 72 selected for the first PCR screening, were such paired sequences and they are listed together and the upstream sequence is listed first. For these ten pairs one PCR primer was placed in each sequence, giving amplification over both sequences. For the 24 sequences that were sequenced in ten dogs, the name used in GenBank is given in parentheses. "$" indicates sequences that here are treated reverse complementary compared to the shotgun sequence found in GenBank.
^b ^PCR primers: P = PCR primer used in PCR screens; O = Outer primer and I = Inner primer in nested amplification; f = forward primer and r = reverse primer.
^c ^Sequencing primers: f = forward primer and r = reverse primer in sequence analysis.
^d ^Analysed positions. Here the region of the original shotgun sequence that was analysed is given. In four cases two separate parts of the shotgun sequence was analysed, because of intervening sequence giving bad quality reads. Click here for file
